# Consolidation of Prospective Memory: Effects of Sleep on Completed and Reinstated Intentions

**DOI:** 10.3389/fpsyg.2016.02025

**Published:** 2017-01-06

**Authors:** Christine Barner, Mitja Seibold, Jan Born, Susanne Diekelmann

**Affiliations:** ^1^Institute of Medical Psychology and Behavioral Neurobiology, University of TübingenTübingen, Germany; ^2^Center for Integrative Neuroscience, University of TübingenTübingen, Germany

**Keywords:** prospective memory, sleep, memory consolidation, future relevance, intention completion, intention reinstatement

## Abstract

Sleep has been shown to facilitate the consolidation of prospective memory, which is the ability to execute intended actions at the appropriate time in the future. In a previous study, the sleep benefit for prospective memory was mainly expressed as a preservation of prospective memory performance under divided attention as compared to full attention. Based on evidence that intentions are only remembered as long as they have not been executed yet (cf. ‘Zeigarnik effect’), here we asked whether the enhancement of prospective memory by sleep vanishes if the intention is completed before sleep and whether completed intentions can be reinstated to benefit from sleep again. In Experiment 1, subjects learned cue-associate word pairs in the evening and were prospectively instructed to detect the cue words and to type in the associates in a lexical decision task (serving as ongoing task) 2 h later before a night of sleep or wakefulness. At a second surprise test 2 days later, sleep and wake subjects did not differ in prospective memory performance. Specifically, both sleep and wake groups detected fewer cue words under divided compared to full attention, indicating that sleep does not facilitate the consolidation of completed intentions. Unexpectedly, in Experiment 2, reinstating the intention, by instructing subjects about the second test after completion of the first test, was not sufficient to restore the sleep benefit. However, in Experiment 3, where subjects were instructed about both test sessions immediately after learning, sleep facilitated prospective memory performance at the second test after 2 days, evidenced by comparable cue word detection under divided attention and full attention in sleep participants, whereas wake participants detected fewer cue words under divided relative to full attention. Together, these findings show that for prospective memory to benefit from sleep, (i) the intention has to be active across the sleep period, and (ii) the intention should be induced in temporal proximity to the initial learning session.

## Introduction

Sleep facilitates the consolidation and subsequent recall of newly encoded memories (Paller and Voss, 2004; Stickgold, 2005; Diekelmann and Born, 2010; Rasch and Born, 2013). Memories that are relevant for future behavior benefit particularly from sleep. Emotional information, for example, is retained better across sleep compared to wake periods than neutral information, with some studies even reporting an additional memory boost for emotional content after sleep at the expense of reduced memory for neutral contents (Payne et al., 2008, 2012, Payne and Kensinger, 2010, 2011). Others found that sleep improves memory consolidation only when subjects expect to be tested on the learned material after sleep, whereas no sleep benefit is evident for memories that are not expected to be tested again (Wilhelm et al., 2011; Van Dongen et al., 2012). When manipulating the relevance of memories by announcing a reward for good performance at testing after sleep, subjects show better performance for a task for which they expected to be rewarded than for a task for which they did not expect any reward, with this difference being only evident after sleep but not after an equivalent interval of wakefulness (Fischer and Born, 2009).

These findings suggest that sleep facilitates memory consolidation selectively if the memory content is regarded as important for the individual and as potentially useful for future actions. Prospective memory is the type of memory that is inherently future-directed, being defined as the ability to execute an intended action at the appropriate time in the future (Ellis, 1996). Scullin and McDaniel (2010) were the first to demonstrate that delayed event-based prospective memory, i.e., the ability to perform an intended action upon detection of a prospective memory cue after a longer time interval, is improved by a period of sleep during the retention interval. In this study, subjects were asked to detect two different cue words, each presented once in three different ongoing tasks after an interval of 12 h either filled with sleep or wakefulness. After the sleep interval, subjects detected the cue words more efficiently compared to the wake period, suggesting that sleep facilitated prospective memory cue detection. In another study by Diekelmann et al. (2013b), using a more naturalistic prospective memory task, subjects were told a cover story, in which they were asked to pay attention that at the test session 2 days later, a vigilance task that they were required to perform was presented in a specific color, which was allegedly a sign for the correct version of the task. Subjects were told that sometimes the experimenter can make a ‘mistake’ and start the wrong task version and in this case, subjects should immediately report the mistake. For subjects who were allowed to sleep after this instruction, the probability to detect the experimenter’s ‘mistake’ at testing was twice as high as for subjects who had stayed awake after formation of the intention (Diekelmann et al., 2013b). A second experiment of this study tested whether the beneficial effect of sleep depended on a specific sleep stage. Higher probabilities to detect the ‘mistake’ were seen after an early slow wave sleep (SWS)-rich sleep period but not after a late rapid eye movement (REM) sleep-rich period, indicating that the beneficial effect of sleep for prospective memory performance is dependent on SWS rather than REM sleep (Diekelmann et al., 2013b). To the best of our knowledge, this is the only study to date examining the role of single sleep stages for prospective memory, and thus, these findings will have to be confirmed in future studies.

Recent evidence further suggests that sleep supports different aspects and processes of prospective remembering (Diekelmann et al., 2013a). Prospective memory includes two sub-components: the ability to remember *that* something has to be done (the prospective component or *intent*), and the ability to remember *what* has to be done (the retrospective component or *content*) (Einstein and McDaniel, 1990, 1996; Kliegel et al., 2008). Moreover, prospective remembering can be accomplished applying either resource-dependent environmental monitoring strategies or automatic spontaneous retrieval processes (McDaniel and Einstein, 2000). According to the monitoring account, attentional resources are needed to keep the intention actively in mind and to search the environment for cues that indicate the correct time and place to execute the intention (Smith, 2003; Smith and Bayen, 2004). Spontaneous retrieval, on the other hand, can occur when the association between the cue and the intended action is strong enough such that the encounter of a cue in the environment automatically brings to mind the associated intention (McDaniel et al., 2004). The ‘dynamic multiprocess framework’ suggests that monitoring and spontaneous retrieval processes interact dynamically to support successful prospective remembering, with one or the other process prevailing depending on the individual, the context and the task demands (Gilbert et al., 2013; Scullin et al., 2013).

A study by Diekelmann et al. (2013a) indicated that sleep after the instruction of an intention improves both the prospective component and the retrospective component of prospective memory and facilitates the use of spontaneous associative retrieval processes to retrieve the intention. In this study, subjects learned 20 cue words, each of which was linked to a specific associated word. After a delay of 2 days, which was filled with a night of sleep or wakefulness and a second (recovery) night of sleep, subjects had to detect the cue words during a lexical decision task, serving as ongoing task, and to type in the associated word upon detecting a cue word. After sleep compared to wakefulness, subjects were more likely to execute the intention, by detecting at least one cue word. Sleep subjects also detected more cue words than wake subjects (prospective component) and remembered more associated words upon cue detection (retrospective component). Interestingly, higher cue detection in sleep subjects was only observed under divided attention conditions when attentional resources were reduced, suggesting that after sleep, subjects were able to rely to a larger extent on spontaneous retrieval processes rather than on attentional monitoring. These findings indicate that sleep strengthens the intentional memory trace and particularly the association between the cue and the associated intention allowing for the automatic activation of the intention upon cue detection.

Building on these findings and based on evidence that sleep preferentially benefits memories that are relevant for future behavior, here we asked whether sleep facilitates intentions only as long as they are active across the retention interval, with the sleep effect vanishing once the intended actions have been completed. In everyday life, it is highly functional to forget or even actively inhibit intentions upon their completion in order to free resources for new plans and intentions as well as to prevent commission errors, i.e., the erroneous execution of intentions that were already executed (Scullin et al., 2012; Walser et al., 2012; Pink and Dodson, 2013; Scullin and Bugg, 2013). For example, inadvertently taking certain medication twice can be highly dangerous for the individual. Once an intention has been realized, the memory for the intended action vanishes, an effect known as the Zeigarnik effect (Zeigarnik, 1927; Mäntylä and Sgaramella, 1997). Upon completion of an intention, the monitoring of the environment for cues that are associated with the intended action, is discontinued (Scullin et al., 2009; Beck et al., 2014), which is associated with the deactivation of brain areas that are engaged in monitoring processes during the active phase of the intention (Beck et al., 2014). Whether the reported effect of sleep on prospective memory is abolished once an intention has been completed is currently unknown. It also remains an open question, whether intentions can be reinstated for sleep-dependent consolidation after their completion. We hypothesized that intentions do no longer benefit from sleep when they are completed before the sleep interval and are thus no longer relevant for future behavior. Additionally, we expected completed intentions to benefit from sleep again when they are reinstated after completion, making them again relevant for later testing. To test these questions, we performed three consecutive experiments, all of which were based on our previously published findings described above (Diekelmann et al., 2013a; from now on called “Basic experiment”).

## Experiment 1: Intention Completed

**FIGURE 1:**
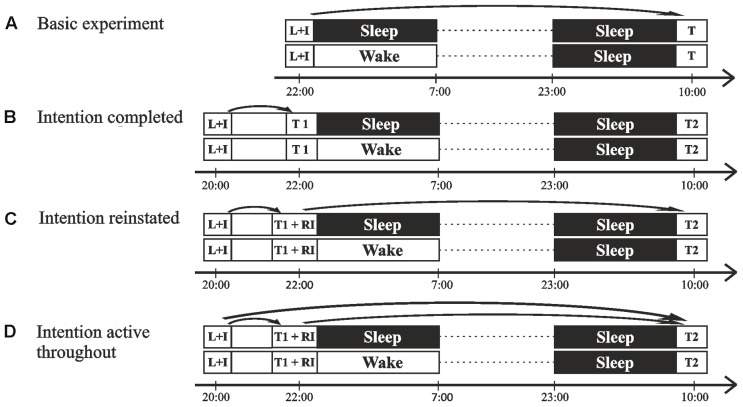
**Experimental design.** **(A)** In our Basic experiment (Diekelmann et al., 2013a), learning (L) and instruction of the intention (I) took place in the evening (∼22.00 h), before a night of sleep (sleep group) or wakefulness (wake group). Subjects were instructed that they would be tested (T) on their prospective memory 2 days later after an additional night of (recovery) sleep. **(B)** In Experiment 1 (Intention completed), learning took place at ∼20.00 h. Thereafter, participants were instructed that they would be tested on their prospective memory 2 h later (T1). Following a night of sleep or wakefulness and another recovery night, a second surprise test took place in the morning (T2). **(C)** In Experiment 2 (Intention reinstated), participants learned and were instructed for the first prospective memory test (T1) 2 h later, like in Experiment 1. After the first test, the intention was reinstated (RI) by instructing subjects that they would be tested on their prospective memory again 2 days later (T2). **(D)** In Experiment 3 (Intention active throughout), learning took place like in Experiments 1 and 2. After learning, subjects were instructed that they would be tested on their prospective memory twice, once in 2 h (T1) and a second time 2 days later (T2). Following the first test, subjects received a reminder instruction (RI) for the second test. Arrows indicate which test session(s) the different instructions are directed at.

**FIGURE 2:**
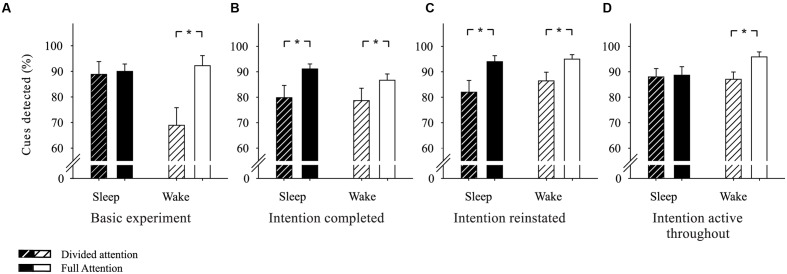
**Effects of sleep on prospective memory.** **(A)** In our Basic experiment (Diekelmann et al., 2013a), sleep participants detected a comparable number of cues under full attention and under divided attention conditions, whereas wake participants were markedly impaired in cue detection under divided attention. **(B)** With the intention completed before sleep in Experiment 1, both sleep and wake subjects showed impaired prospective memory performance, i.e., diminished numbers of cues detected, under divided attention. **(C)** Reinstatement of the intention after its completion in Experiment 2 did not suffice to reinstate the sleep benefit. Both sleep and wake subjects were impaired in cue detection under divided attention. **(D)** When the intention was active throughout the entire experimental period in Experiment 3, i.e., when subjects expected both test sessions from the beginning, sleep benefitted prospective memory despite the first completion of the intention before sleep. While wake subjects detected less cues under divided attention compared to full attention, sleep subjects were not impaired by divided attention. Means ± SEM are shown. ^∗^*p* < 0.05.

Our Basic experiment (Diekelmann et al., 2013a) established that sleep facilitates the ability to execute an intended action at the appropriate time after a delay of 2 days (see **Figure 1A** for the experimental design). Most interestingly, subjects who were allowed to sleep after intention formation detected more cues in the ongoing task at the delayed test session specifically under divided attention conditions. Sleep and wake subjects performed equally well in cue detection when they had full attentional resources available. With reduced attentional resources, however, cue detection was markedly impaired in wake subjects but remained completely unaffected in sleep subjects (see **Figure 2A**), suggesting that sleep strengthened the cue-intention association thereby favoring spontaneous retrieval processes. However, from these data it remains unclear whether the sleep effect is specific for the memories associated with the intention or whether sleep simply non-selectively strengthens memories in the associative network that were encoded shortly before sleep. Here we manipulated the intentional status of the memories by having the intended action completed before the sleep interval. Specifically, we asked whether the beneficial effect of sleep vanishes when the intended behavior is completed before sleep and thus, the intention is no longer relevant.

**FIGURE 3:**
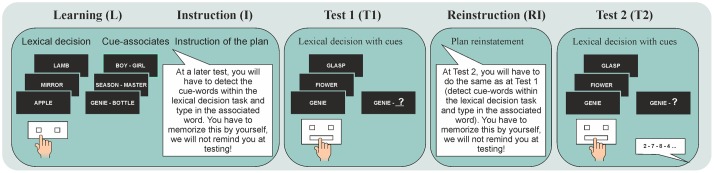
**Prospective memory task.** In all experiments, subjects took part in a learning session, during which they practiced on the lexical decision task and learned 20 cue-associate word pairs. The instruction of prospective memory (I) differed for the different experiments. In Experiment 1 (Intention completed), subjects were instructed that at a test session in 2 h (Test 1), some of the 20 cue words could occur within the lexical decision task and if they recognized a cue word they should press the ‘space’ bar and type in the respective associated word. Subjects were explicitly told that they need to memorize this instruction because the experimenter would not remind them of what to do at the test session. After Test 1, subjects did not receive another reinstruction (RI) but Test 2 took place 2 days later as a surprise test. Test 2 was identical to Test 1, except that in order to manipulate available attentional resources, subjects performed a secondary task in parallel (monitoring spoken digits for two consecutive even digits) either during the first or second half of the lexical decision task. In Experiment 2 (Intention reinstated), participants followed the same protocol as in Experiment 1 (Intention completed), with the only difference that after Test 1, subjects received a reinstruction of the intention (RI) in which they were told that they would have to perform on the task again in a second test session 2 days later (Test 2). In Experiment 3 (Intention active throughout), the protocol was identical to that of Experiment 2 (Intention reinstated), with the only exception that during the initial instruction (I), subjects were told that they would have to perform on the task twice, in test session 1 after 2 h and in test session 2 after 2 days. Like in Experiment 2 (Intention reinstated), they received an additional reinstruction (RI) for Test 2 after completion of Test 1. In our Basic experiment (Diekelmann et al., 2013a), participants were tested for their prospective memory only once after 2 days (Test 2), with this test being instructed (I) immediately after the learning session.

Subjects performed on the same task with the same instruction as in the Basic experiment (Diekelmann et al., 2013a) (**Figure 3**). However, a first test session took place in the evening 2 h after instruction of the prospective memory task, before one group of subjects went to sleep (*n* = 18) whereas the other stayed awake the following night (*n* = 15; **Figure 1B**). During the first test session, subjects already completed the intention. To ensure that the intention was no longer active during subsequent sleep, participants were told immediately after this test session that they *would not* have to do this task again. Nevertheless, a second test session occurred unexpected to the subjects 2 days later, like in the Basic experiment. Based on evidence indicating that sleep selectively strengthens memories of relevance for future behavior (Fischer and Born, 2009; Wilhelm et al., 2011), we hypothesized that the improving effect of sleep on prospective memory would disappear when the intended behavior is completed before sleep. Specifically, sleep should no longer facilitate the storage of the cue-intention association, thus, both sleep and wake subjects would be expected to rely to a larger extent on monitoring processes and should therefore to the same extent be impaired in cue detection under divided attention.

### Methods

#### Participants

A total of 33 subjects (19 females, mean age [±SD]: 21.94 ± 2.97), with regular sleep-wake cycles (≥ 6 h sleep per night) and no shift work for at least 6 weeks prior to the experiments participated in Experiment 1. Subjects reported no history of any neurological, psychiatric or endocrine disorder and did not take any medication at the time of the experiments. Ingestion of caffeine and alcohol was not allowed from the day before until the end of the experiments and subjects were instructed to stay awake during the day after the sleep/wake night. Prior to the experimental night, subjects spent one adaptation night in the sleep laboratory. All subjects gave written informed consent and were paid for participation. The study was approved by the local ethics committee of the University of Lübeck.

#### Design and Procedure

All subjects reported to the laboratory at 19:30 h, filled in questionnaires, underwent the initial learning session at 20:00–20:45 h and received the prospective memory instruction thereafter (**Figure 1B**). Subjects then watched a non-disturbing movie until they were informed about whether they were assigned to the sleep or the wake group. In the sleep condition, electrodes were attached for standard polysomnographic recordings, including electroencephalogram (at sites C3 and C4), electrooculogram and electromyogram. Polysomnographic recordings were visually scored offline according to standard criteria (Rechtschaffen and Kales, 1986). The first test session took place between 22:00 and 22:30 h. In the sleep condition, subjects then went to bed for regular sleep between 23:00 and 07:00 h, whereas subjects in the wake condition stayed awake throughout the night, spending the time with reading, watching TV or playing simple games. Subjects in both conditions left the laboratory the next morning. After spending the day awake and another night of sleep at home, allowing the subjects in the wake condition to recover from their initial sleep loss, they returned to the laboratory for the second test session at 10:00 h the following day. Subjects kept record of their activities and their bedtime and wake-up time for the night of sleep at home.

#### Prospective Memory Task

The same prospective memory task as in our Basic experiment (Diekelmann et al., 2013a) was applied. Participants were required to detect cues (i.e., specific cue words) and perform associated actions (i.e., recall associated second words) in an ongoing task (i.e., lexical decision task; **Figure 3**). In the initial learning session, subjects first practiced the lexical decision task (serving as ongoing task later) without any prospective memory cues. Subjects were presented in a random sequence with 100 word stimuli, half of which were existing German words. The other half were ‘non-words’ which were derived from German words by substituting one consonant (Marsh et al., 2002, 2003). Subjects were instructed to press as fast and as accurately as possible the right key (on a keyboard) for correct words and the left key for non-words (with the respective index finger). After practice on the lexical decision task, subjects learned 20 cue-associate word pairs for the subsequent prospective memory task. Half of the word pairs were semantically related, e.g., *Genie – Bottle*, and half were not semantically related, e.g., *Season – Master* (however, since semantic relatedness did not affect memory measures differentially for the sleep and wake group, related and unrelated word pairs were combined in all analyses). Subjects learned the cue words first separately from the associated words. Cue words were presented successively for 5 s each with 1-s breaks in between. After presentation of all cue words, subjects recalled the words in a free recall test. Presentation and free recall was repeated to a criterion of 90% (i.e., 18) correctly recalled cue words to ensure that all subjects would perfectly recognize the cue words in a recognition test and prospective memory retrieval would not depend on how well cue words had been learned (see also Diekelmann et al., 2013a). The 90% criterion in the free recall test was chosen based on pilot studies indicating that this criterion produced practically perfect performance on the word recognition test. After learning of cue words, subjects learned the respective associated words for each cue word. Word pairs were presented successively for 5 s each with a 1-s break in between. For each word pair, the cue word was presented on the left side of the screen and the associated word on the right side of the screen. After all word pairs had been presented once, a cued recall test followed in which subjects upon presentation of each cue word were required to press first the ‘space’ bar, then a field opened on the screen where they should type in the corresponding associated word. After pressing ‘enter,’ the next cue word appeared without feedback about the correctness of the previous response. If subjects did not achieve the criterion of 60% (i.e., 12) correctly recalled associated words, all word pairs were presented again for re-learning, followed by another cued recall test. Presentation of the word pairs and cued recall was repeated until subjects met the 60% learning criterion. The 60% criterion was chosen based on previous studies indicating maximal effects of sleep on consolidation of word pair memories at this criterion (Drosopoulos et al., 2007).

The prospective memory instruction was given after the learning phase. Subjects were informed that, apart from testing their lexical discrimination abilities, we were also interested in their ability to remember to do something in the future. For this purpose, some of the cue words they had just learned would occasionally appear within the lexical decision task when they would be tested again. Subjects were instructed that the test would take place 2 h later that same evening. They were instructed that when they detected a cue word within the lexical decision task at this test they should press the ‘space’ bar and then a field would open where they should type in the associated word, confirm with ‘enter’ and continue with the lexical decision task. Subjects had to repeat this instruction in their own words to ensure full understanding. They were explicitly instructed to memorize this instruction because at the test session the experimenter would *not* remind them of what to do.

In the test session 2 h later, subjects performed the lexical decision task without being reminded of the instructed intention. The lexical decision task during testing contained 390 word stimuli, i.e., 185 real words, 185 non-words, and the 20 learned cue words. Cue words were presented every 16th to 20th word (mean: 18th). A break was made after half of the words had been presented. After this first test session subjects were told that during the second test session after 2 days they would have to perform on completely different tasks for another part of the study. Thus, they would not have to do the prospective memory task again. The second test session, however, was basically identical to the first test session. Yet, subjects were explicitly instructed in the second test session to detect the cue words and type in the associated words, as they did not expect another test of the previous task. Note that this procedure in fact corresponds to the standard assessment of prospective memory where subjects are explicitly instructed before testing and prospective memory performance is quantified by the subject’s ability to detect cues during a distracting ongoing task. In order to test whether subjects used a relatively resource-demanding monitoring strategy or a relatively resource-independent spontaneous-associative retrieval strategy, we directly manipulated available attentional resources during the second test session: during one of the halves of the task (balanced across subjects) the subjects performed in parallel an auditory attention task in which spoken digits were presented via loudspeakers at a rate of one digit every 2 s. The subjects were required to press a separate key whenever two even digits occurred consecutively.

#### Control Tasks

In the end of the two test sessions, i.e., after the first test in the evening as well as after the second test 2 days later, memory for the cue words was tested in a recognition test. The 20 cue words were presented randomly mixed with 40 distractor words (not presented before) and subjects had to indicate for each word if it was a cue word or new. Additionally, memory for the associated words was tested in a cued recall. Each cue word was presented on the screen and subjects had to recall the respective associated word. No feedback was given on whether or not their response was correct. To control for general alertness and vigilance, all subjects performed on a vigilance task for the duration of 5 min before learning and after the two test sessions. In this task, a dot randomly appeared at the left or right side of a computer screen every 2–10 s and participants had to respond as quickly as possible by pressing the corresponding left or right button. Because vigilance data were missing for two subjects in the wake group and from one subject in the sleep group, the available sample size added up to *n* = 17 (sleep group) and *n* = 13 (wake group) for the analyses of this task. Subjects also rated their subjective sleepiness on the Stanford Sleepiness Scale before learning and after the test sessions, ranging from 1 (“feeling active, vital, alert, or wide awake”) to 7 (“no longer fighting sleep, sleep onset soon; having dream-like thoughts”) (Hoddes et al., 1973). Furthermore, after the second test session, all subjects completed a questionnaire to assess rehearsal of the prospective memory instruction and rehearsal of the cue-associate pairs during the retention interval as well as the use of strategies to remember the instruction and the cue-associates. For the final cue recognition test and the cued recall of the associated words, one data set of the wake group was missing, thus analyses included *n* = 14 participants in the wake group and *n* = 18 participants in the sleep group.

#### Statistical Analysis

All variables were analyzed using analyses of variance (ANOVA) and *post hoc t*-tests. Additionally, non-parametric *post hoc* tests (i.e., Mann–Whitney *U* Test and Wilcoxon-Test) were used when deviations from the normal distribution occurred. Level of significance was set to *p* = 0.05. Greenhouse–Geisser correction for degrees of freedom was applied where appropriate.

### Results

#### Prospective Memory Task Performance

With the intention completed before sleep, sleep did no longer improve the prospective component of prospective memory at testing after 2 days. At the second test session, subjects in the sleep group detected 91.11 ± 1.96% of the cue words without the secondary task and 80.00 ± 4.64% with the secondary task to be performed in parallel (*z* = 2.55, *p* = 0.01, *d* = 0.76). Wake subjects detected 86.67 ± 3.47% and 78.67 ± 4.87% (*z* = 2.39, *p* = 0.02, *d* = 0.51) of the cue words without and with the secondary task, respectively (main effect ‘with/without secondary task’: *F*(1,31) = 14.00, *p* < 0.001, $\eta_{p}^{2}$ = 0.31; main effect ‘sleep/wake’: *F*(1,31) = 0.35, *p* = 0.56; ‘sleep/wake’ × ‘with/without secondary task’ interaction: *F*(1,31) = 0.37, *p* = 0.55; **Figure 2B**). Thus as expected, divided attention by the secondary task during the second test session impaired cue detection in the sleep group to the same extent as in the wake group.

Completing the intention before sleep also prevented the beneficial effect of sleep on the retrospective component of prospective memory observed in the Basic experiment (see **Supplementary Figure 1A**). Relative to the number of cues detected, sleep subjects remembered 63.90 ± 3.89% of the associated words and wake subjects remembered 68.51 ± 4.26%, *F*(1,31) = 0.64, *p* = 0.43 (for main effect ‘sleep/wake,’ **Supplementary Figure 1B**) at the second test, which was independent of attentional resources available, *F*(1,31) = 0.01, *p* = 0.92, for the interaction ‘sleep/wake’ × ‘with/without secondary task’; *F*(1,31) = 0.46, *p* = 0.50, for main effect ‘with/without secondary task.’

Initial learning performance of cue words and associated words was comparable between the sleep and wake group. Subjects in the sleep and wake group remembered 19.00 ± 0.20 and 18.73 ± 0.18 cue words in the criterion learning trial, *U* = 111.00, *z* = -0.93, *p* = 0.38, and needed on average 2.33 ± 0.20 and 2.33 ± 0.19 trials to reach the criterion, *U* = 131.50, *z* = -0.14, *p* = 0.92. Recall of associated words was 15.83 ± 0.54 and 15.67 ± 0.60 in the criterion learning trial, *t*(31) = 0.21, *p* = 0.84, with a mean of 1.22 ± 0.13 and 1.13 ± 0.09 learning trials, *U* = 129.50, *z* = -0.32, *p* = 0.86, in the sleep and wake group, respectively. Likewise, performance during the first completion of the prospective memory task in the evening was comparable between the sleep and wake group. Sleep and wake participants detected 84.72 ± 4.84% and 86.67 ± 2.87% of cue words, *U* = 128.50, *z* = -0.24, *p* = 0.82, and they remembered 69.25 ± 3.79% and 64.90 ± 3.86% of associated words relative to the number of correctly detected cue words, *t*(31) = 0.80, *p* = 43.

#### Ongoing Task Performance

Sleep and wake subjects did not differ in lexical decision task performance at learning, at the first test in the evening and at the second test after 2 days [**Table 1**; trials without the secondary task for reaction time: main effect ‘sleep/wake’: *F*(1,31) = 0.00, *p* = 0.97, main effect ‘learning/test1/test2’: *F*(1.66,51.54) = 5.75, *p* = 0.008, interaction ‘sleep/wake’ × ‘learning/test1/test2’: *F*(1.66,51.54) = 0.28, *p* = 0.72; for error rate: main effect ‘sleep/wake’: *F*(1,31) = 0.92, *p* = 0.35, main effect ‘learning/test1/test2’: *F*(1.62,50.06) = 2.48, *p* = 0.11, interaction ‘sleep/wake’ × ‘learning/test1/test2’: *F*(1.62,50.06) = 3.19, *p* = 0.06]. Sleep and wake subjects both responded significantly faster at the second test in comparison to the learning session and the first test in the evening [learning vs. second test: *t*(32) = 2.50, *p* = 0.02, *d* = 0.25; first test vs. second test: *t*(32) = 3.29, *p* < 0.01, *d* = 0.34; learning vs. first test: *t*(32) = -1.25, *p* = 0.22], while the error rate did not change over time.

**Table 1:** Ongoing task performance.

	Intention completed		Intention reinstated		Intention active throughout	
			
	Sleep	Wake	Sleep	Wake	Sleep	Wake
**Reaction time**					
Learning	1215 ± 71	1238 ± 52	1081 ± 47	1006 ± 59	979 ± 55	1229 ± 66
Test 1	1285 ± 67	1294 ± 64	1200 ± 48	1064 ± 53	1090 ± 55	1279 ± 75
Test 2						
Full attention	1159 ± 64	1119 ± 73	1109 ± 52	1021 ± 58	1016 ± 54	1222 ± 59
Divided attention	1580 ± 70	1537 ± 70	1367 ± 33	1421 ± 74	1420 ± 84	1610 ± 66
**Error rate**					
Learning	4.83 ± 0.57	6.07 ± 1.26	4.54 ± 1.03	4.86 ± 0.86	5.80 ± 1.01	4.36 ± 0.75
Test 1	4.74 ± 0.63	3.73 ± 0.44	3.90 ± 0.72	4.45 ± 0.41	5.11 ± 0.71	4.39 ± 0.72
Test 2						
Full attention	3.23 ± 0.47	5.28 ± 1.01	3.65 ± 0.60	4.33 ± 0.71	4.94 ± 1.04	3.66 ± 0.80
Divided attention	4.51 ± 0.42	6.27 ± 1.21	5.48 ± 0.77	6.30 ± 1.07	5.39 ± 0.85	5.75 ± 1.23


*Reaction times (in ms) and error rates (in %) in the lexical decision task during learning, test 1, and test 2. For test 2, measures are provided separately for full attention and divided attention. Means ± SEM are shown.*

In the second test session, performing the secondary auditory attention task in parallel slowed down reaction times for lexical decisions in both sleep subjects and wake subjects [for main effect ‘with/without secondary task’: *F*(1,31) = 198.20, *p* < 0.001, $\eta_{p}^{2}$ = 0.87, for ‘sleep/wake’ main effect: *F*(1,31) = 0.20, *p* = 0.66, for ‘sleep/wake’ × ‘with/without secondary task’: *F*(1,31) = 0.00, *p* = 0.97], and increased error rates [for main effect ‘with/without secondary task’: *F*(1,31) = 6.89, *p* = 0.013, $\eta_{p}^{2}$ = 0.18, for main effect ‘sleep/wake’: *F*(1,31) = 3.37, *p* = 0.08, for ‘sleep/wake’ × ‘with/without secondary task’: *F*(1,31) = 0.10, *p* = 0.75], confirming that the secondary auditory attention task put a high load on attentional resources.

#### Control Tasks

The final cue recognition test at the end of the second test session confirmed that both sleep and wake participants had almost perfect retrospective memory for the cue words (recognition accuracy: sleep, 99.07 ± 0.34%; wake, 97.86 ± 0.77%; *U* = 97.00, *z* = -1.25, *p* = 0.24). Moreover, sleep and wake participants did not differ in cued recall of the associated words [sleep: 64.44 ± 3.61%, wake: 67.86 ± 4.15%; *t*(30) = -0.62, *p* = 0.54]. Sleep and wake subjects were overall comparable in their performance on the vigilance task regarding reaction time (for main effect ‘sleep/wake’ *p* = 0.78, for interaction ‘sleep/wake’ × ‘learning/test1/test2’ *p* = 0.97, for main effect ‘learning/test1/test2’ *p* < 0.01, $\eta_{p}^{2}$ = 0.23) and error rate (for main effect ‘sleep/wake’ *p* = 0.78, for interaction ‘sleep/wake’ × ‘learning/test1/test2’ *p* = 0.26, for main effect ‘learning/test1/test2’ *p* = 0.80) as well as in subjective sleepiness (for main effect ‘sleep/wake’ *p* = 0.13, for main effect ‘learning/test1/test2’ *p* < 0.001, $\eta_{p}^{2}$ = 0.44, for interaction ‘sleep/wake’ × ‘learning/test1/test2’ *p* = 0.21), despite generally lower vigilance performance (all *p* < 0.04) and higher sleepiness ratings at the first test in the evening (*p* < 0.001) for all subjects irrespective of group (**Table 2**). Subjects in the sleep group also displayed normal sleep patterns during the night following the first test session (**Table 3**).

**Table 2:** Vigilance and subjective sleepiness.

	Intention completed		Intention reinstated		Intention active throughout	
			
	Sleep	Wake	Sleep	Wake	Sleep	Wake
**Vigilance performance**					
***Reaction time***					
Learning	348 ± 12	345 ± 10	412 ± 13	418 ± 11	420 ± 12	426 ± 9
Test 1	359 ± 13	355 ± 11	421 ± 14	446 ± 13	419 ± 12	434 ± 8
Test 2	337 ± 11	331 ± 10	406 ± 10	424 ± 11	405 ± 12	426 ± 7
***Error rate***					
Learning	4.41 ± 0.73	4.62 ± 0.84	3.5 ± 0.64	3.93 ± 0.63	1.83 ± 0.45	2.03 ± 0.57
Test 1	3.82 ± 0.94	4.42 ± 0.58	3.5 ± 0.64	3.57 ± 0.97	2.50 ± 0.65	2.34 ± 0.66
Test 2	5.00 ± 0.91	3.46 ± 0.67	3.17 ± 0.57	3.04 ± 0.54	2.33 ± 0.86	2.50 ± 0.82
**Subjective Sleepiness**					
Learning	2.33 ± 0.20	2.00 ± 0.20	2.93 ± 0.23	2.21 ± 0.30	2.53 ± 0.26	2.47 ± 0.15
Test 1	3.56 ± 0.25	2.87 ± 0.26	3.93 ± 0.25	5.50 ± 1.50	4.07 ± 0.25	3.29 ± 0.22
Test 2	2.11 ± 0.21	2.07 ± 0.18	2.27 ± 0.21	2.86 ± 0.29	1.93 ± 0.12	3.00 ± 0.27


*Vigilance performance (reaction times in ms and error rates in % of all trials) and subjective sleepiness (Stanford Sleepiness Scale) during learning, test 1 and test 2. There were no significant differences between respective groups in both experiments. Means ± SEM are shown.*

**Table 3:** Sleep parameters.

Sleep stage	Intention completed	Intention reinstated	Intention active throughout
Sleep time	447.81 ± 8.03	446.43 ± 10.69	439.33 ± 8.60
W	6.94 ± 1.50	8.60 ± 3.07	13.37 ± 2.60
S1	19.69 ± 2.61	20.85 ± 2.45	27.87 ± 4.12
S2	233.89 ± 6.20	240.30 ± 8.75	237.70 ± 8.69
SWS	79.50 ± 6.71	76.93 ± 5.84	72.03 ± 7.60
REM	104.56 ± 4.16	108.30 ± 6.19	101.03 ± 6.09


*Sleep time (total sleep time), W (wake), S1 (sleep stage 1), S2 (sleep stage 2), SWS (slow wave sleep, i.e., the sum of sleep in stages 3 and 4 sleep) and REM (rapid eye movement sleep) in minutes. Means ± SEM are shown.*

### Discussion

As expected, Experiment 1 showed that a period of sleep following an already completed intention does not improve the ability to implement the behavior when participants are asked to perform the prospective memory task again 2 days later. As hypothesized, participants in both the sleep and wake group detected significantly less cues when their attention was reduced by a secondary auditory task compared to the full attention condition, supporting the notion that both sleep and wake subjects relied to a greater extent on resource-intensive monitoring rather than spontaneous retrieval for cue detection. We suggest that after completing the intention, with the knowledge that the intended actions do not have to be performed again, sleep no longer fosters the storage of the associations between the cues and the intended actions in the associative memory network, such that after sleep, subjects rely to a lesser extent on automatic activation of the intention upon encounter with the cues. These findings are consistent with the Zeigarnik effect demonstrating that memories of uncompleted actions are better retained than memories of already completed actions (Zeigarnik, 1927; Mäntylä and Sgaramella, 1997). In combination with our Basic experiment, which showed a sleep effect for uncompleted intentions (Diekelmann et al., 2013a), these results indicate that an intention, for profiting from sleep, needs to be active over the sleep period and thus, needs to be relevant for future behavior.

## Experiment 2: Intention Reinstated

Experiment 1 showed that sleep no longer facilitates the ability to execute an intended action after a delay of 2 days when the intention has already been completed before the night of sleep or wakefulness. In everyday life, however, completed intentions can become relevant again. For example, a person might form the intention to water the flowers in her flat. After completing this intention, she might renew the intention to water the flowers again 2 days later. This raises the question, whether it is possible to reinstate a completed intention to make it sensitive for sleep-dependent consolidation processes again. In Experiment 2, we examined the effect of sleep on intentions that were completed and then reinstated before sleep or wakefulness.

Subjects performed on the same task with the same instruction as in Experiment 1, i.e., subjects completed the intention 2 h after the initial intention formation. However, after the first test session the intention was reinstated by instructing the subjects that they would have to do the task again at a second test session 2 days later (**Figures 1C** and **3**). Following this instruction, one group of subjects went to sleep (*n* = 15) whereas the other group stayed awake (*n* = 14) the following night like in Experiment 1. We hypothesized that the improving effect of sleep on prospective memory performance would reappear with the intention being reinstated before sleep. Specifically, sleep should again facilitate the storage of the cue-intention associations, such that after sleep, subjects would be expected to rely to a larger extent on spontaneous retrieval and should be less impaired in cue detection under divided attention compared to wake subjects.

### Methods

#### Participants

A total of 29 healthy young adults (19 females, mean age [±SD]: 22.69 ± 2.98), were included in the analysis of Experiment 2. Criteria for subjects to participate in the study were as in Experiment 1. In total, four sleep subjects and nine wake subjects had to be excluded. Nine participants were excluded due to problems with the protocol (three participants talked about the experiment, three participants did not detect any of the cue words in the first test session, one participant slept for 2.5 h during the day after the experimental night, one participant got sick during the experimental night, and one participant exceeded the pre-defined body-mass-index cut-off of 25). Four outliers had to be excluded due to very poor prospective memory performance during the second test (more than 2 SD below the overall mean). All subjects gave written informed consent and were paid for participation. The study was approved by the local ethics committee of the University Tübingen.

#### Design and Procedure

The experimental design and procedure was identical to Experiment 1, with the only exception that after the first test session in the evening, subjects were instructed, that they would have to complete the task again 2 days later and that they would have to keep this instruction in mind because the experimenter would not remind them of what to do at the second test session (**Figure 1C**).

#### Prospective Memory Task

Tasks and materials were identical to Experiment 1 (intention completed) except that subjects after the first test session were instructed about the second test 2 days later (**Figure 3**). Although subjects expected the second test by then, they were still explicitly instructed before the second test session to detect the cue words and to type in the associated words in the lexical decision task, in order to ensure comparable conditions with Experiment 1.

Control tasks and statistical analyses were as for Experiment 1.

### Results

#### Prospective Memory Task Performance

Reinstating the intention after completion of the task before sleep did not suffice for sleep to improve cue detection in the prospective memory task. Subjects in the sleep group detected 94.00 ± 2.35% of the cue words without the secondary task and 82.00 ± 4.60% with the secondary task to be performed in parallel (*z* = -2.57, *p* = 0.01, *d* = 0.88). Wake subjects detected 95.00 ± 1.74% and 86.43 ± 3.41% (*z* = -2.17, *p* = 0.03, *d* = 0.88) of cue words without and with the secondary task, respectively [main effect ‘with/without secondary task’: *F*(1,27) = 16.51, *p* < 0.001, $\eta_{p}^{2}$ = 0.38; main effect ‘sleep/wake’: *F*(1,27) = 0.50, *p* = 0.49; ‘sleep/wake’ × ‘with/without secondary task’ interaction: *F*(1,27) = 0.46, *p* = 0.50; **Figure 2C**]. Thus, divided attention impaired cue detection in the sleep group to the same extent as in the wake group, similar to Experiment 1, suggesting that reinstating the intention before sleep did not make the intention subject to sleep-dependent consolidation processes again.

Reinstating the completed intention also did not affect the sleep benefit on the retrospective component of prospective memory. Relative to the number of cues detected, sleep subjects at the second test remembered 71.61 ± 4.42% of the associated words and wake subjects remembered 74.56 ± 4.58%, *F*(1,27) = 0.22, *p* = 0.65 (for main effect ‘sleep/wake,’ **Supplementary Figure 1C**), which was independent of attentional resources available, *F*(1,27) = 0.01, *p* = 0.94 for the interaction ‘sleep/wake’ × ‘secondary task’ and *F*(1,27) = 0.16, *p* = 0.69 for main effect ‘with/without secondary task.’

As in Experiment 1, learning performance of cue words and associated words was comparable between groups. Subjects in the sleep and wake group remembered 18.47 ± 0.17 and 18.71 ± 0.22 cue words in the criterion learning trial (*U* = 89.00, *z* = -0.78, *p* = 0.45), and needed on average 2.53 ± 0.19 and 2.71 ± 0.30 trials to reach the criterion (*U* = 96.00, *z* = -0.39, *p* = 0.72). Recall of associated words was 15.93 ± 0.71 and 17.00 ± 0.55 in the criterion learning trial, *t*(27) = -1.18, *p* = 0.25, with a mean of 1.27 ± 0.12 and 1.36 ± 0.13 learning trials, *U* = 95.50, *z* = -0.52, *p* = 0.70, in the sleep and wake group, respectively. During the first completion of the task in the evening, subjects were comparable in prospective memory task performance. Sleep participants detected 82.33 ± 2.84% of cue words and wake participants detected 85.36 ± 3.53% (*U* = 79.00, *z* = -1.16, *p* = 0.26). Relative to the number of correctly detected cue words, both groups were also comparable in the number of remembered associated words [sleep: 67.58 ± 4.81%, wake: 75.50 ± 3.44%; *t*(27) = -1.32, *p* = 0.20].

#### Ongoing Task Performance

Sleep and wake subjects did not differ in lexical decision performance at learning, at the first test as well as at the second test [**Table 1**; without the secondary task: main effect ‘sleep/wake’ for reaction time *F*(1,27) = 2.13, *p* = 0.16, for error rate *F*(1,27) = 0.33, *p* = 0.57, interaction ‘sleep/wake’ × ‘learning/test1/test2’ for reaction time *F*(1.54,41.57) = 0.76, *p* = 0.44, for error rate *F*(1.50,40.49) = 0.07, *p* = 0.89]. Like in Experiment 1, reaction times changed across time, independent of sleep and wake conditions [main effect ‘learning/test1/test2’ *F*(1.54,41.57) = 6.08, *p* = 0.009, $\eta_{p}^{2}$ = 0.18]. All subjects slowed down responses from the learning to the first test session and accelerated their reaction time again from the first to the second test session [learning vs. first test: *t*(28) = -4.84, *p* < 0.001, *d* = 0.45; first test vs. second test: *t*(28) = 2.35, *p* = 0.03, *d* = 0.34; learning vs. second test: *t*(28) = -0.72, *p* = 0.48]. Error rates did not change over time [main effect ‘learning/test1/test2’ *F*(1.50,40.49) = 1.12, *p* = 0.32].

Performing the secondary auditory attention task in parallel during the second test session slowed down reaction times for lexical decisions in both sleep subjects and wake subjects [for main effect ‘with/without secondary task’: *F*(1,27) = 76.28, *p* < 0.001, $\eta_{p}^{2}$ = 0.74; for ‘sleep/wake’ main effect: *F*(1,27) = 0.06, *p* = 0.80, for interaction ‘with/without secondary task’ × ‘sleep/wake’: *F*(1,27) = 3.49, *p* = 0.07], and increased error rates [for main effect ‘with/without secondary task’: *F*(1,27) = 31.07, *p* < 0.001, $\eta_{p}^{2}$ = 0.54; for main effect ‘sleep/wake’: *F*(1,27) = 0.49, *p* = 0.49, for interaction ‘with/without secondary task’ × ‘sleep/wake’: *F*(1,27) = 0.05, *p* = 0.83].

#### Control Tasks

The final cue recognition test after the second test session confirmed, like in Experiment 1, that sleep and wake participants almost perfectly recognized all of the cue words (recognition accuracy: sleep 99.00 ± 0.56%, wake 99.52 ± 0.21%, *U* = 98.00, *z* = -0.38, *p* = 0.87). Memory for the associated words in the cued recall was also comparable in the sleep and wake group [sleep 72.33 ± 4.28%, wake 78.57 ± 3.69%, *t*(27) = 1.10, *p* = 0.28]. Like in Experiment 1, sleep and wake subjects were also comparable in performance on the vigilance task (reaction times and error rates) as well as in reported sleepiness during learning and both test sessions (for main effect ‘sleep/wake’ and interaction ‘sleep/wake’ × ‘learning/test1/test2’: all *p* > 0.10, **Table 2**), despite generally slower reaction times and higher sleepiness ratings at the first test in the evening for all subjects [for main effect ‘learning/test1/test2’: reaction time *p* < 0.001, $\eta_{p}^{2}$ = 0.29; sleepiness *p* = 0.003, $\eta_{p}^{2}$ = 0.27; error rate *p* = 0.60]. Subjects in the sleep group also displayed normal sleep patterns during the night following the first test session (**Table 3**).

### Discussion

Experiment 2 examined the possibility to reinstate completed intentions for a sleep-dependent improvement. Contrary to our hypothesis, a period of sleep following intention reinstatement did not facilitate the ability to execute the intended action after a delay of 2 days. Like in Experiment 1, participants in both the sleep group and the wake group detected less cues in the ongoing task when their attention was reduced by the secondary auditory attention task, suggesting that both groups relied to a larger extent on monitoring and sleep subjects were not able to recruit on less resource-dependent spontaneous retrieval processes to detect cue words.

This finding indicates that reinstating an intention after its completion does not make the intentional memory trace gain access to sleep-dependent memory processing. Instructing participants to do the task again 2 days later, with this instruction being provided only after having completed the task, does not seem to be sufficient to reinstate the intention for the enhancing effects of sleep. One possible explanation for this failure is that the reinstatement took place too long after the original learning experience. The execution of intentions has been suggested to depend on a link formed between the intention and the context in which the intention is expected to be executed (Marsh et al., 2006), with this context effect being most evident when the intention-context link is formed during initial encoding of the intention (Nowinski and Dismukes, 2005). Similarly, Scullin and McDaniel (2010) observed a sleep effect on prospective memory only in the context, i.e., the ongoing task, which was temporally paired with the prospective memory instruction during the learning session. Scullin and McDaniel (2010) argued that the intention-context association is strengthened by consolidation processes during sleep, which then facilitates subsequent spontaneous retrieval processes. Based on this evidence, it can be speculated that in the present paradigm the intention must be formed in close proximity to the initial learning session in order for sleep to strengthen the memory representations of the cue-associate relations.

## Experiment 3: Intention Active Throughout

Experiments 1 and 2 established that (i) sleep no longer benefits the execution of intentions when these intentions are already completed before sleep, and (ii) instructing subjects for the second test session after completion of the intention in the first test session is not sufficient to reinstate the sleep benefit. Importantly, in Experiments 1 and 2, the prospective memory instruction given after the learning session was only directed at the first test session 2 h after learning, but this instruction never included the second test session 2 days later. In Experiment 2, the reinstatement of the intention took place after the first test session, that is, about 2 h after the end of the initial learning session and the initial prospective memory instruction. Considering that sleep might act to strengthen the intentional cue-associate connection that is formed in the learning context, 2 h of time difference between the reinstatement of the intention and the initial encoding of the cue-associates might have been too long in order to link the renewed intention to the previously learned cue-associate word pairs. Accordingly, in Experiment 3 we tested whether completed intentions benefit from sleep if the subjects are instructed about both test sessions (the first one after 2 h and the second one after 2 days) immediately after the learning session, such that the intention for the second delayed test is formed in temporal proximity to the cue-associate learning context and is active throughout the entire experimental period.

The same task and setup was used as in Experiments 1 and 2. However, after the learning session, subjects were instructed that they would have to do the task 2 days later, with this delayed test session being introduced as the main part of the experiment. In addition, subjects were told that they would have to complete the task once already in 2 h, for practice purposes. After the first test session, the instruction for the second test session was repeated to keep the procedure comparable with Experiment 2 (**Figure 1D**). After this instruction, one group of subjects went to sleep (*n* = 15) whereas the other stayed awake the following night (*n* = 17). We expected that with the intention active across the entire retention interval and the intention being formed in close proximity to the initial learning, sleep would strengthen the intentional association between the cues and the associated actions. Therefore, we hypothesized that after sleep, subjects would rely to a larger extent on spontaneous retrieval and would be less impaired in cue detection under divided attention conditions compared to wake subjects.

### Methods

#### Participants

A total of 32 healthy young adults (16 females, mean age [±SD]: 22.91 ± 2.72) were included in the analysis of Experiment 3. Inclusion and exclusion criteria were identical to Experiments 1 and 2. Overall 4 participants of the sleep group and 2 participants from the wake group had to be excluded. One participant showed pathological sleep with a REM sleep-onset latency of 5.5 min and one participant slept for 2 h during the day after the experimental night. Four outliers had to be excluded due to poor prospective memory performance during the second test session (more than 2 SD below the overall mean). All subjects gave written informed consent and were paid for participation. The study was approved by the local ethics committee of the University Tübingen.

#### Design and Procedure

The experimental design and procedure were identical to Experiment 1, with the only exception that this time, immediately after the learning session, subjects were instructed that there would be two test sessions during which they would have to complete the instructed intention (**Figure 1D**).

#### Prospective Memory Task

Tasks and materials were identical to Experiments 1 and 2, except that subjects after the learning session were instructed about both test sessions (**Figure 3**). With this instruction, they were told that they would have to detect the cue words and type in the associated words at the test session in 2 days and for practice purposes also in 2 h before the night of sleep or wakefulness. They were instructed that for both test sessions they would have to keep this instruction in mind because the experimenter would not remind them of what to do. Although subjects expected the second test, they were still explicitly instructed before the second test session to detect the cue words and type in the associated words in the lexical decision task, in order to ensure comparable conditions with Experiments 1 and 2.

#### Control Tasks

Control tasks were identical to Experiments 1 and 2. Because vigilance data were missing for one subject in the wake group, the available sample size was *n* = 15 (sleep group) and *n* = 16 (wake group) for the analyses of the vigilance task. For the final cue recognition test and the cued recall of the associated words, data of one subject in the sleep group was missing, thus analyses included *n* = 14 participants in the sleep group and *n* = 17 participants in the wake group for these data.

Statistical analyses were as in Experiments 1 and 2.

### Results

#### Prospective Memory Task Performance

As expected, with the intention instructed immediately after the learning session, sleep improved the detection of cued words in the lexical decision task under divided attention conditions. Subjects in the sleep group detected 88.67 ± 3.36% of cue words without the secondary task and 88.00 ± 3.27% with the secondary task to be performed in parallel (*z* = -0.38, *p* = 1). Wake subjects, on the other hand differed in cue detection when they had to perform the secondary task in parallel. They detected 95.88 ± 1.93% of cues without and 87.06 ± 2.68% with the secondary task [*z* = -2.28, *p* = 0.02; ‘sleep/wake’ × ‘with/without secondary task’ interaction: *F*(1,30) = 4.58, *p* = 0.04, $\eta_{p}^{2}$ = 0.13; main effect ‘sleep/wake’: *F*(1,30) = 0.81, *p* = 0.38; main effect ‘with/without secondary task’: *F*(1,30) = 6.21, *p* = 0.02, $\eta_{p}^{2}$ = 0.17; **Figure 2D**].

Although descriptively on a higher level, the retrospective component of prospective memory was not significantly improved by the sleep manipulation. Relative to the number of cues detected, sleep subjects at the second test remembered 71.01 ± 3.97% of the associated words and wake subjects remembered 66.55 ± 3.73%, *F*(1,30) = 0.67, *p* = 0.42 (for main effect ‘sleep/wake,’ **Figure 1D**), which was independent of attentional resources available, [*F*(1,30) = 2.54, *p* = 0.12 for the interaction ‘sleep/wake’ × ‘secondary task’ and *F*(1,30) = 0.02, *p* = 0.90 for the main effect ‘with/without secondary task’).

As in Experiments 1 and 2, learning performance of cue words and associated words was comparable between groups. Subjects in the sleep and wake group remembered 19.00 ± 0.22 and 18.59 ± 0.21 cue words in the criterion learning trial (*U* = 92.00, *z* = -1.44, *p* = 0.175), and needed on average 2.33 ± 0.19 and 2.71 ± 0.29 trials to reach the criterion (*U* = 105.00, *z* = -0.96, *p* = 0.35). Recall of associated words was 15.73 ± 0.67 and 15.00 ± 0.66 in the criterion learning trial (*U* = 104.00, *z* = -0.90, *p* = 0.38) with a mean of 1.27 ± 0.12 and 1.35 ± 0.12 learning trials (*U* = 116.50, *z* = -0.52, *p* = 0.71) in the sleep and wake group, respectively. During the first completion of the task in the evening, sleep and wake participants did not differ in prospective memory performance. Sleep subjects detected 84.00 ± 3.69% of cue words and wake subjects detected 87.65 ± 4.87% (*U* = 96.00, *z* = -1.22, *p* = 0.23). Relative to the number of correctly detected cue words, sleep and wake subjects remembered 68.82 ± 3.69% and 68.25 ± 3.83% of the associates, *t*(30) = 0.11, *p* = 0.92.

#### Ongoing Task Performance

In the lexical decision task, subjects in the sleep group showed overall faster reaction times than the wake group [**Table 1**; main effect ‘sleep/wake’: *F*(1,30) = 6.94, *p* = 0.01, $\eta_{p}^{2}$ = 0.19], which was consistent across learning and both test sessions [interaction ‘learning/test1/test2’ × ‘sleep/wake’: *F*(2,60) = 0.89, *p* = 0.42]. Additionally, independent of sleep and wake conditions, subjects slowed down in their reaction time from the learning to the first test session [*t*(31) = -3.48, *p* < 0.01] and showed faster reaction times again from the first to the second test session (*z* = -2.67, *p* < 0.01), while the learning session and the second test session did not differ [*z* = -0.69, *p* = 50; main effect ‘learning/test1/test2’: *F*(2,60) = 6.59, *p* = 0.003, $\eta_{p}^{2}$ = 0.18]. Error rates in the lexical decision task did not differ between groups [for main effect ‘sleep/wake’: *F*(1,30) = 1.26, *p* = 0.27; for main effect ‘learning/test1/test2’: *F*(1.39,41.80) = 1.08, *p* = 0.33; for interaction ‘sleep/wake’ × ‘learning/test1/test2’: *F*(1.39,41.80) = 0.26, *p* = 0.69].

Performing the secondary auditory attention task in parallel during the second test session slowed down reaction times for lexical decisions in both sleep subjects and wake subjects [for main effect ‘with/without secondary task’: *F*(1,30) = 182.12, *p* < 0.001, $\eta_{p}^{2}$ = 0.86; for interaction ‘with/without secondary task’ × ‘sleep/wake’: *F*(1,30) = 0.07, *p* = 0.79], again with sleep subjects overall responding faster [main effect ‘sleep/wake’: *F*(1,30) = 4.90, *p* = 0.04, $\eta_{p}^{2}$ = 0.14]. Divided attention by the secondary task also increased the error rates in the sleep group as well as in the wake group [for main effect ‘with/without secondary task’: *F*(1,30) = 8.03, *p* < 0.01, $\eta_{p}^{2}$ = 0.21, for main effect ‘sleep/wake’: *F*(1,30) = 0.12, *p* = 0.74, for interaction ‘with/without secondary task’ × ‘sleep/wake’: *F*(1,30) = 3.31, *p* = 0.08].

#### Control Tasks

Like in Experiments 1 and 2, the final cue recognition test at the second test session confirmed that sleep and wake participants almost perfectly remembered all of the cue words (recognition accuracy: sleep 99.81 ± 0.41%, wake 99.41 ± 0.20%, *U* = 95.50, *z* = -1.07, *p* = 0.32). Sleep and wake participants were also comparable in final cued recall of the associated words [sleep 73.21 ± 3.65%, wake 70.29 ± 3.22%, *t*(29) = -0.60, *p* = 0.55]. Likewise, sleep and wake subjects did not differ in their performance in the vigilance task (for reaction time and error rate: main effects ‘sleep/wake’ and interactions ‘sleep/wake’ × ‘learning/test1/test2’ *p* > 0.30, **Table 2**), despite generally slower reaction times for all subjects at the first test session in the evening, like in Experiments 1 and 2 (main effect ‘learning/test1/test2’: for reaction time *p* = 0.03, $\eta_{p}^{2}$ = 0.21; for error rate *p* = 0.61). The sleep and wake groups differed in subjective sleepiness at the first test session (*U* = 72.00, *z* = -2.19, *p* = 0.03) and the second test session (*U* = 52.00, *z* = -3.11, *p* < 0.01), with the sleep participants being more sleepy at test 1 (sleep: 4.07 ± 0.24, wake: 3.29 ± 0.23) and less sleepy at test 2 (sleep: 1.93 ± 0.23, wake: 3.00 ± 0.21). Sleepiness levels at learning were comparable between groups [*U* = 125.50, *z* = -0.09, *p* = 0.93; interaction: ‘learning/test1/test2’ × ‘sleep/wake’: *F*(2,60) = 12.68, *p* < 0.001, $\eta_{p}^{2}$ = 0.30; main effect: ‘learning/test1/test2’: *F*(2,60) = 28.14, *p* < 0.001, $\eta_{p}^{2}$ = 0.48; main effect: ‘sleep/wake’: *F*(1,30) = 0.12, *p* = 0.74]. Finally, subjects in the sleep group displayed normal sleep patterns during the night following prospective memory instructions (**Table 3**).

### Discussion

In accordance with our hypothesis, Experiment 3 showed that sleep benefits the ability to execute an intention that has been completed once before sleep, when subjects are instructed about the delayed prospective memory test immediately after the initial learning session. Thus, sleep facilitates the delayed execution of the intention if the intention is formed in close temporal proximity to the learning of the cue-associates and if subjects know from the beginning that they have to execute the intention again 2 days later. In this case, relative to performance under full attention, sleep subjects were not impaired in cue detection under divided attention conditions. Wake subjects, on the other hand, differed in performance under divided attention and full attention, with a relatively lower performance when the attention was reduced. This pattern of results suggests that sleep subjects were able to rely to a larger extent on spontaneous retrieval processes to detect the cues, while wake subjects depended more on attention-based monitoring strategies. With the intention being formed in close proximity to the learning session, sleep presumably strengthened the link between the intention and the cue-associate representations in the memory network allowing for an automatic activation of the intention upon encountering the cue words.

The finding that wake participants generally showed slower reaction times in the lexical decision task was unexpected. Importantly, this difference was evident across all sessions, i.e., wake subjects already performed slower during the learning session and the first test session in the evening, excluding the possibility that slower reaction times were due to the wakefulness manipulation. Sleepiness is unlikely to explain the differences in lexical decision reaction times because wake subjects showed slower reaction times throughout all sessions but only displayed lower sleepiness than the sleep group at the first test session and higher sleepiness at the second test session. Moreover, sleepiness was not significantly correlated with reaction times in the lexical decision task, neither at test 1 (sleep: *r* = 0.43, *p* = 0.11, wake: *r* = -0.30, *p* = 0.25) nor at test 2 (sleep: full attention, *r* = 0.03, *p* = 0.91, divided attention, *r* = -0.15, *p* = 0.61; wake: full attention, *r* = 0.07, *p* = 0.79, divided attention, *r* = 0.15, *p* = 0.58). Importantly, sleepiness at the second test session did also not significantly correlate with the number of cues detected under full attention (sleep: *r* = -0.50, *p* = 0.06, wake: *r* = 0.21, *p* = 0.42) as well as under divided attention (sleep: *r* = -0.40, *p* = 0.15, wake: *r* = 0.25, *p* = 0.33). Finally, reaction times in the lexical decision task were not associated with the number of cues detected at test 1 (sleep: *r* = -0.04, *p* = 0.90, wake: *r* = 0.09, *p* = 0.73) and at test 2 (sleep: full attention, *r* = 0.32, *p* = 0.24, divided attention: *r* = 0.48, *p* = 0.07; wake: full attention, *r* = 0.43, *p* = 0.09, divided attention, *r* = -0.17 *p* = 0.52), indicating that sleepiness and reaction times in the lexical decision task did not affect the number of cues detected.

On a descriptive level, wake subjects in Experiment 3 overall performed very well in cue detection, such that in the full attention condition their performance was above the average of around 90%, and in the divided attention condition they performed on a level comparable with the sleep participants. Although this difference was not significant (all *p* > 0.07), it is in contrast to our Basic experiment (Diekelmann et al., 2013a) where under divided attention wake participants performed significantly worse than sleep participants. It could be speculated that this high performance level in wake subjects was due to subjectively higher sleepiness in these participants, which might have led to overcompensation with regard to the detection of the cue words, with wake subjects focusing all available attentional resources on the prospective memory task. However, subjective sleepiness was not correlated with the number of cues detected, speaking against this possibility. Moreover, objective alertness levels as measured by reaction times and error rates in the vigilance task were not different in wake subjects compared to sleep participants. Vigilance task performance was also not correlated with cue detection in the prospective memory task (all *p* > 0.05), speaking against the possibility that prospective memory performance was affected by general alertness levels. Alternatively, higher overall performance in wake participants might be interpreted in light of recent findings showing that sleep deprivation can be a state of heightened plasticity due to prefrontal disinhibition (Sprenger et al., 2015). In the wake group, disinhibition under sleep deprivation following the first test session might have triggered plastic changes during subsequent recovery sleep, leading to overall higher performance levels at the second test 2 days later.

## Cross-Experiment Comparisons

For a *post hoc* comparison of cue detection across all four experiments, an ANOVA with the between-subjects factors ‘experiment’ [Basic experiment/Experiment 1 (Intention completed)/Experiment II (Intention reinstated)/Experiment III (Intention active throughout)] and ‘sleep/wake’ and the within-subject factor ‘with/without secondary task’ was conducted. This overall comparison confirmed that cue detection for sleep and wake participants differed depending on the instructed intention and on whether participants had to perform the prospective memory task under full attention or divided attention conditions [interaction ‘experiment’ × ‘sleep/wake’ × ‘with/without secondary task’: *F*(3,112) = 3.65, *p* = 0.02, $\eta_{p}^{2}$ = 0.09].

When analyzing all sleep groups separately, the beneficial effect of sleep on cue detection differed across experiments [interaction ‘experiment’ × ‘with/without secondary task’: *F*(3,61) = 2.60, *p* = 0.06, $\eta_{p}^{2}$ = 0.11; main effect ‘experiment’: *p* = 0.80, main effect ‘with/without secondary task’: *p* < 0.01]: the sleep effect, as reflected in comparable cue detection under full attention and divided attention, was evident only when the intention was induced together with the previous encoding of cue-associates and when the intention was active throughout the entire retention period (comparison of the Basic experiment and Experiment 3: *p* > 0.70 for interaction ‘experiment’ × ‘with/without secondary task’ and main effect ‘with/without secondary task’). No such sleep effect was evident when participants completed the intention before sleep and when the intention was simply reinstated after completion, as reflected in decreased cue detection when participants performed the task under divided attention compared to full attention [comparison of Experiment 1 and Experiment 2: *F*(1,31) = 17.33, *p* < 0.001, $\eta_{p}^{2}$ = 0.36, for main effect ‘with/without secondary task,’ *F*(1,31) = 0.03, *p* = 0.87, for interaction ‘experiment’ × ‘with/without secondary task’]. Comparing the sleep groups of the intention active throughout experiment (Experiment 3, sleep effect) with the sleep groups of the intention completed and the intention reinstated experiments (Experiments 1 and 2, no sleep effect), confirmed a significantly better cue detection under divided attention with the intention active throughout the retention period [interaction ‘experiment’ × ‘with/without secondary task’: *F*(1,31) = 4.88, *p* = 0.04, $\eta_{p}^{2}$ = 0.14 for Experiment 3 vs. Experiment 1, and *F*(1,28) = 7.63, *p* = 0.01, $\eta_{p}^{2}$ = 0.21 for Experiment 3 vs. Experiment 2]. Similarly, the sleep groups of the Basic experiment tended to perform better in cue detection under divided attention than the sleep groups of the intention completed and the intention reinstated experiments [interaction ‘experiment’ × ‘with/without secondary task’: *F*(1,33) = 2.65, *p* = 0.11, $\eta_{p}^{2}$ = 0.07 for Basic experiment vs. Experiment 1, and *F*(1,30) = 3.25, *p* = 0.08, $\eta_{p}^{2}$ = 0.10 for Basic experiment vs. Experiment 2].

Separate analyses of all wake groups across the four experiments showed a decrease in cue detection under divided attention compared to full attention in all experiments (comparison across all experiments and when comparing single experiments with each other: all *p* < 0.005 for main effects ‘with/without secondary task,’ all *p* > 0.11 for interaction effects ‘experiment’ × ‘with/without secondary task’). Overall performance in cue detection also differed across experiments (*p* = 0.03 for main effect ‘experiment’), with *post hoc* tests, comparing the single experiments, showing that wake subjects in Experiment 3 performed better than in the Basic experiment and in Experiment 1 (*p* = 0.01 and *p* = 0.04) and wake subjects in Experiment 2 performed better than in the Basic experiment and in Experiment 1 (*p* = 0.03 and *p* = 0.09; all other *p* > 0.70).

Despite these differences between experiments in the wake groups, the cross-experiment comparisons are in line with the reported findings of the single experiments and confirm the beneficial effect of sleep for intentions selectively and only when the intention is active during sleep and when it is induced in temporal proximity to the learning session.

## General Discussion

Based on the previous finding from our Basic experiment that sleep benefits prospective memory (Diekelmann et al., 2013a), here we investigated in three experiments, whether this beneficial effect of sleep depends on the relevance of prospective memories for actions to be performed in the future. Particularly, we asked whether the sleep effect is abolished when the intended action is completed before sleep and whether an intention can be reinstated for a sleep benefit after its completion. We show in Experiment 1 that once an intention is completed before sleep, the subsequent execution of prospective memory is no longer facilitated by a night of sleep. In Experiment 2, we found that contrary to our hypothesis, reinstructing the intention after its completion is not sufficient to reinstate the intention for a sleep benefit. Experiment 3 finally demonstrates that sleep improves intention execution if the intention for both test sessions is formed in temporal proximity to the initial learning session and thus, subjects expect right from the start to be tested on the prospective memory task again after its first completion.

These findings are in line with previous evidence indicating that sleep-dependent memory consolidation is selective in the way that it particularly fosters memories that are relevant for future behavior. Sleep has been shown to favor the consolidation of memories that are expected to be rewarded at testing as compared with memories for which reward was not expected (Fischer and Born, 2009). Likewise, emotionally charged memories benefit to a larger extent from sleep than neutral memories (Payne et al., 2008, 2012; Payne and Kensinger, 2010, 2011), and sleep selectively consolidates memories for which subjects expect to be tested after sleep, whereas memories that are not expected to be tested do not benefit from sleep (Wilhelm et al., 2011; Van Dongen et al., 2012). In accord with this evidence, Experiment 1 demonstrates for the first time that completing an intention before sleep abolishes the previously shown beneficial effect of sleep on the subsequent execution of the intention. These findings are reminiscent of the Zeigarnik effect in suggesting that sleep improves prospective memories only as long as they still have to be executed in the future, i.e., as long as they are relevant for future behavior, with sleep’s supportive effect vanishing once the intention has been completed. They corroborate the concept of prospective memory as a fundamentally dynamic type of memory existing only in the presence of specific (driving) intentions.

Based on our Basic experiment (Diekelmann et al., 2013a) we suggest that sleep improves prospective remembering by strengthening the association between the prospective memory cue and the associated intended action, thereby favoring spontaneous associative retrieval processes upon encountering the prospective memory cue after sleep (McDaniel et al., 2004; Scullin and McDaniel, 2010; Diekelmann et al., 2013a). Experiment 1 indicates that this proposed strengthening of the cue-action association is dependent on the intention being active across the sleep period. Considering the results of our Basic experiment (Diekelmann et al., 2013a), it could be argued that sleep did not specifically improve the intentional aspect of prospective memory but rather non-specifically strengthened the associations encoded prior to sleep, thereby indirectly facilitating spontaneous retrieval of the intention as a by-product of ‘simple’ retrospective associative memory consolidation. However, if this was true, we would have expected a beneficial effect of sleep also for completed intentions, considering that the cue-associates were encoded similarly prior to sleep with and without completion of the intention. The finding that, despite similar retrospective memory encoding, sleep did not benefit completed intentions suggests that – similar to the Zeigarnik effect – the effect of sleep for prospective memory critically depends on the intention being active across sleep, i.e., the intention being relevant for the successful execution of future actions.

There are different possible explanations for the abolished sleep effect after intention completion. Zeigarnik attributed better memory for uncompleted intentions on the ‘tension’ that is generated when a task is interrupted or not yet completed (Zeigarnik, 1927). According to this assumption, uncompleted intentions in the present study might have existed in a state of increased tension, with this tension possibly signaling importance of the memory, similar to an emotional charge, which then leads to a preferential access to sleep-dependent memory consolidation. With the intention completed before sleep, the tension might be released and, with it, the relevance signal for consolidation during sleep.

More contemporary theories suggest that intentions that are to be executed in the future show a privileged status of heightened activation over information that is simply to be remembered (Goschke and Kuhl, 1993; Marsh et al., 1998), an effect known as the ‘intention superiority effect’. In this vein, uncompleted intentions that are still to be executed might show heightened activation making these intentions relevant and susceptible to sleep-dependent consolidation, whereas reduced or abolished activation after intention completion might in turn reduce their sensitivity to consolidation processes during sleep. It has been shown that finished intentions no longer elicit longer reaction times when cues are presented again, whereas cues of suspended intentions that are still relevant for later execution elicit slower responses (Scullin et al., 2009, 2011). Likewise, subjects can more efficiently deactivate intentions and thereby avoid commission errors when the intention is completed than when it is not yet completed (Bugg and Scullin, 2013). However, the exact mechanisms of this process, e.g., whether the heightened activation of intentions passively fades after their completion or whether completed intentions become actively inhibited, are not yet fully understood (see for example Bugg et al., 2013; Pink and Dodson, 2013; Scullin and Bugg, 2013; Walser et al., 2013).

An alternative explanation is based on the idea that the intentional memory does not only comprise the cue-action association but additionally includes a link to a (separate) intention representation that together with the cue-action association forms an intentional memory network. The intention representation, by signaling future relevance, might tag the cue-action association for consolidation processes during sleep. In order for the intentional network to be efficiently established, the intention representation might have to be encoded together with the cue-action association in order to tag this association for subsequent consolidation. This idea is in line with evidence suggesting that a link formed between the intention and the context in which the intention has to be executed determines subsequent prospective memory performance in that same context (Nowinski and Dismukes, 2005; Marsh et al., 2006). Moreover, it has been shown that sleep particularly facilitates intention execution in the context that was temporally paired with the encoding of the intention (Scullin and McDaniel, 2010). Thus, in Experiment 1, the initial encoding of the cue-action associations might have been specifically linked to the expected execution of the intention at the first retest 2 h later, with this link therefore not being subject to subsequent sleep-dependent consolidation processes.

The findings of Experiment 2 and Experiment 3 are in line with this latter explanation. Experiment 2 shows that it is not possible to reinstate a completed intention for sleep-dependent consolidation simply by announcing, after completion of the intention, that subjects would have to execute the task again 2 days later. While a renewed tension according to the Zeigarnik effect as well as a renewed activation according to the ‘intention superiority effect’ could be expected to be achieved relatively easy with a reinstruction of the intention, it might be hard or even impossible to connect the intention to the original cue-action associations after the completion of the intention. With the reinstruction of the intention taking place about 2 h after the initial encoding of the cue-action associations, the reinstructed intention representation might not be effectively connected to the cue-action associations, thus failing to tag these associations for consolidation processes during sleep. Experiment 3 shows that the sleep effect reappears when subjects are instructed right after the initial learning of the cue-action associations that they would be tested on their prospective memory twice. This finding suggests that the temporal proximity between intention formation and encoding of the cue-action associations is a key factor for the sleep benefit to emerge, which is in line with evidence from Scullin and McDaniel (2010) showing that intentions benefit from sleep particularly when they are temporally paired with the context in which they have to be executed later on, but not when there is a temporal gap between the intention instruction and the context encounter.

On a neurophysiological level, it can be speculated that the intention instruction given shortly after initial learning, i.e., in the learning context, recruits neuronal plastic processes that are initiated during encoding, such that the cue-action associations can be tagged by the intention for further processing during sleep. There is ample evidence that newly encoded retrospective memories become reactivated during periods of subsequent sleep (for review, see Abel et al., 2013; Rasch and Born, 2013). Neuronal network activity that is evident during the encoding of new experiences is re-expressed (‘replayed’) in a similar way during sleep following the learning experience (Wilson and McNaughton, 1994; Nádasdy et al., 1999; Ji and Wilson, 2007). Such replay events are assumed to strengthen the underlying neuronal connections and have mainly been observed during SWS. A recent study found sleep-dependent improvements of prospective memory specifically after SWS-rich sleep periods (Diekelmann et al., 2013b), suggesting that similar replay mechanisms might be involved in the reprocessing of prospective memories during sleep. In the wake state, successful prospective memory has been shown to rely mainly on activations in prefrontal cortical areas (Burgess et al., 2003, 2007), and there is evidence that sleep-associated memory reactivation extends from hippocampal to cortical areas including prefrontal cortex (Euston et al., 2007; Peyrache et al., 2009; Igloi et al., 2015). Reactivations in prefrontal areas during sleep might be specifically linked to intentional memory networks to support prospective memory and planning (Buhry et al., 2011; Schwindel and McNaughton, 2011). In the present study, the intention instruction given shortly after the initial learning experience might have tagged the newly encoded intentional memory networks for subsequent reactivation during sleep, and presumably during SWS, to strengthen the intentional cue-action associations. The execution of the intention during the expected completion of the task, on the other hand, might not (or to a lesser extent) induce the plastic processes on which a reinstatement of the intention/tag could build. With no (or a weaker) reinstatement of the intention, the intentional memory network might not be tagged for subsequent replay during sleep, or alternatively, the replay might not be strong enough to induce sufficient strengthening of the underlying connections. Future studies should scrutinize potential neurophysiological mechanisms of intention formation and intention reinstatement systematically.

Importantly, Experiment 3 excludes the possibility that the mere execution of the task before sleep abolishes the sleep effect. The fact that Experiment 3 shows a beneficial effect of sleep on prospective remembering despite prior task execution strongly argues against the possibility that simply performing on the task before sleep explains the lacking sleep effect in Experiment 2. In showing a beneficial sleep effect on prospective memory, Experiment 3 also replicates and extends findings from our Basic experiment (Diekelmann et al., 2013a) using an identical relatively complex prospective memory task including 20 different cue words. Together with earlier findings reporting sleep benefits for a simple one-item prospective memory task in a more real world-like setting (Diekelmann et al., 2013b) and a more typical laboratory prospective memory task using two cue words (Scullin and McDaniel, 2010), there is now convincing evidence that sleep facilitates prospective remembering in a range of different tasks and settings.

Overall performance levels of cue detection were higher in the experiments of the present study compared to our Basic experiment (Diekelmann et al., 2013a), with this effect being most pronounced in the wake groups. The overall higher performance levels can be explained by the additional test session before sleep introduced in all three experiments. Detecting the cue words and remembering the associates during the first test session before sleep might have served as an additional practice of the cue-associate pairs. The observation that overall cue detection further increased across experiments in the wake groups could be explained by the increasing number of intention instructions and execution of the task. Previous evidence indicates that the perceived importance of the prospective memory task affects cue detection performance, with higher importance typically leading to better cue detection (McDaniel and Einstein, 2000; Walter and Meier, 2014, 2016). Considering that in the present study, additional (re-)instructions were introduced in each of the successive experiments, this might have inadvertently added further importance to the prospective memory task, increasing relevance in the intentional memory network. We can only speculate why this increase in overall cue detection across experiments was more pronounced in wake subjects. Sleep possibly supports different mechanisms of prospective memory performance, such as memory consolidation and reactivation, which might ‘overwrite’ processes otherwise affecting prospective memory performance in the wake state, such as additional practice and repeated intention instructions. By strengthening the intentional memory connections, we assume that sleep favors spontaneous-associative retrieval processes (Diekelmann et al., 2013a), whereas wake participants rely to a larger extent on monitoring strategies, with the latter possibly being more susceptible to the manipulation of task importance (McDaniel and Einstein, 2000). Future studies should directly test these ideas.

While the generally enhanced performance level might have been still sufficient to yield a sleep benefit for the prospective component of prospective memory, i.e., cue detection, the intentional memories might have been too strong for a sleep benefit on the retrospective component to occur. At first glance, this is surprising considering that a successful sleep effect on prospective memory could be expected to entail a benefit for both the prospective component to detect the cue words and the retrospective component to remember the associated words, as was evident in our Basic experiment (Diekelmann et al., 2013a). However, even though subjects did not receive any feedback on their performance during the first test session, there is evidence that memory retrieval *per se* can strengthen retrospective memories (Roediger and Karpicke, 2006; Karpicke and Roediger, 2008; Smith et al., 2013). Moreover, it has been suggested that sleep benefits retrospective memory optimally when memories are encoded with medium strength, whereas memories that are too strong or too weak do not benefit from sleep (Stickgold, 2009; Wilhelm et al., 2012). Thus, stronger cue-action associations following the first prospective memory test in the evening before sleep might have hindered the emergence of an improving effect of sleep on the retrospective component. Additionally, the retrospective component was not affected by divided attention in any of the experiments – other than the prospective component, which was impaired under divided attention conditions. This pattern of results replicates the findings from our Basic experiment (Diekelmann et al., 2013a) and is in line with evidence indicating that retrospective memory recall is less dependent on attentional resources (Craik et al., 1996; Iidaka et al., 2000), particularly with the retrospective component (word recall) and the secondary task (digit monitoring) relying on different processing systems in our paradigm (i.e., verbal vs. numerical) (Fernandes and Guild, 2009; Skinner et al., 2009).

Altogether, our four experiments show that prospective memory benefits from a night of sleep only if the intention is active throughout the entire experimental period, suggesting that sleep facilitates prospective memory only as long as the intention is relevant for future actions and if the intention is formed in close proximity to the initial learning session. The sleep effect on prospective memory is abolished once the intention has been completed before sleep and cannot be reinstated by simply reinstructing the intention after its completion. Future studies will have to examine the neuronal mechanisms underlying these effects, including the potential role of different sleep stages and sleep parameters.

## Ethics Statement

Experiment 1 was approved by the ethics committee of the University of Lübeck. Experiments 2 and 3 were approved by the ethics committee of the medical faculty of the University of Tübingen. Participants received written and oral information of the study before they gave their written consent.

## Author Contributions

CB designed the research, performed the research, analyzed the data, and wrote the paper. MS performed the research and analyzed the data. JB designed the research and wrote the paper. SD designed the research, analyzed the data, and wrote the paper.

## Conflict of Interest Statement

The authors declare that the research was conducted in the absence of any commercial or financial relationships that could be construed as a potential conflict of interest.
