# Supplementary material for: Consolidation of Prospective Memory: Effects of Sleep on Completed and Reinstated Intentions
Source: Front Psychol. 2017 Jan 6;7:2025. doi: 10.3389/fpsyg.2016.02025 (PMC5216900; doi:10.3389/fpsyg.2016.02025)
Supplement: Supplementary file 1 [file Data_Sheet_1.docx]

Supplementary Material

**Consolidation of prospective memory:
Effects of sleep on completed and reinstated intentions**

Christine Barner*, Mitja Seibold, Jan Born, Susanne Diekelmann

*** Correspondence:** Christine Barner: Christine.barner@uni-tuebingen.de


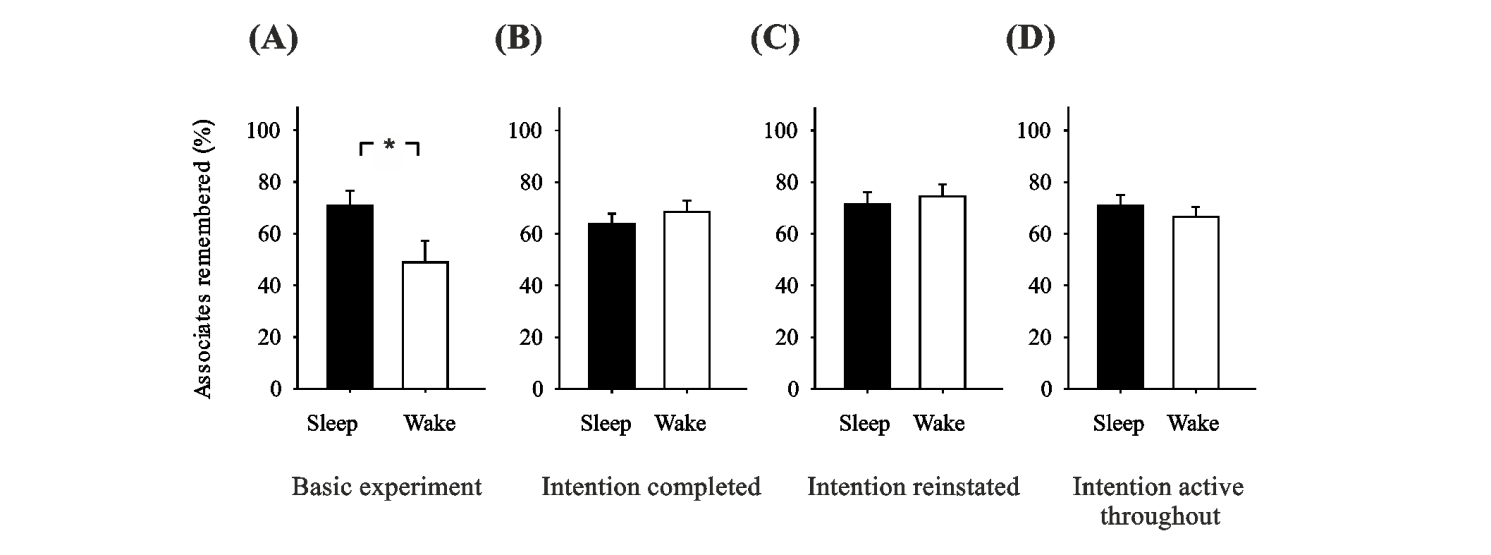


**Supplementary Figure 1.** **Percentage of associated words** relative to the number of correctly detected cue words (measuring the retrospective component) in **(A)** the Basic experiment (Diekelmann, 2013a) and the three experiments of the present study: **(B)** Intention completed, **(C)** Intention reinstated, and **(D)** Intention active throughout. Sleep and wake participants´ performance were comparable in the three experiments of the present study, whereas sleep subjects remembered more associates than wake subjects in the Basic experiment (Diekelmann, 2013a). * *p* < 0.05
